# Mal de Debarquement Syndrome: A Rare Entity—A Case Report and Review of the Literature

**DOI:** 10.1155/2015/918475

**Published:** 2015-08-05

**Authors:** Veronica Nwagwu, Rakesh Patel, Jerome Okudo

**Affiliations:** ^1^Geriatric and Palliative Medicine Division, Internal Medicine Department, University of Michigan Health System, 4260 Plymouth Road, Ann Arbor, MI 48109, USA; ^2^Family Medicine Department, Saint Mary Mercy Hospital, 36475 Five Mile, Livonia, MI 48154, USA; ^3^School of Public Health, University of Texas, 1200 Pressler Street, Houston, TX 77030, USA

## Abstract

Mal de Debarquement Syndrome (MDS) is a rare, understudied, underdiagnosed, and self-limiting condition. Etiology and incidence are unknown. It is characterized by abnormal sensation of motion/balance reported after travel by air, land, and sea; being reexposed to motion/activity relieves it. Symptoms may last from minutes to years. Workup though required reveals no findings; it is a diagnosis of exclusion. While no efficacious treatment exists, amitriptyline and benzodiazepines as well as supportive therapy have proved to be useful. We have described a 40-year-old Caucasian female who presented for the evaluation of persistent rocking and swaying sensation after a ship cruise which lasted for one week. Patient was treated with benzodiazepines after extensive workup and is now stable. A high index of suspicion is required to make a diagnosis.

## 1. Introduction

Mal de Debarquement Syndrome (MDS) is a rare condition, which presents with motion (bobbing, rocking, or swaying) after a trip (boat, car, train, or plane), and it is usually noticed upon return to the ground [1–20]. Patients feel a sense of unsteadiness. There is significant relief upon return to motion [2, 3, 5, 6]. Helmet-wearing stimulated virtual setting can also trigger MDS [16]. It can last for hours, months, or years [2]. After its first description in modern medical literature in 1987, there has not been significant research for MDS [7, 19]. Typically, there is motion adaptation but symptoms may last long enough and there may be no long standing morbidity [6]; however what is consistent in the medical literature is that there is no definitive time frame in the description of the symptoms of this condition [1–20]. While some of the patients recover, a sizeable proportion does not recover [14]. There has been confusion between motion sickness and MDS; however, in MDS, the patient while in motion has no symptoms [13, 14, 16]. On physical and neurological examination and laboratory evaluation of the MDS patient, there will be no findings [1–20]. MDS is a diagnosis of exclusion [5]. While many of the patients are women [1–6], a few have been men [7]. Benzodiazepines and amitriptyline have given some degree of relief to the patients [1, 5, 18]. In addition, patients may require reassurance [6]. It has been shown that MDS patients make several visits to the physician before an accurate diagnosis is made and this is because many physicians do not recognize the symptoms or confuse it with its differentials [7, 14]. The aim of this case report is to create awareness in the medical community, to increase the indices of suspicion, to promote early diagnosis, and to generate more research in this area.

## 2. Case Presentation

A 40-year-old Caucasian female with a past medical history of hypertension presented to the outpatient clinic for evaluation of abnormal perception of motion which she described as constant rocking and swaying after a ship cruise which lasted for one week. It began few hours after returning from a week ship cruise four months ago. The symptoms are worse at bedtime when lying still and early in the morning upon awakening. Symptoms were alleviated by motion activities like driving and being in a moving car. She denied nausea and vomiting. She had no history of headaches, double vision, vertigo, ringing in the ears, or hearing deficits. In addition to her past medical history of hypertension for which she takes lisinopril, she had acid reflux and cholecystectomy. She denied tobacco dependence and alcohol or illicit drug abuse. Physical examination was unremarkable, with no signs of depression elicited. The patient had not responded to empirical treatment with various motion sickness therapies, which included meclizine, scopolamine, low dose steroid, and Medrol for possible labyrinthitis. Videonystagmography (VNG) and Vestibular Evoked Myogenic Potential (VEMP) to determine the functionality of the ears, vestibular deficits, and integrity of the inner ear were all normal. The ENT and neurology team evaluated her. All her laboratory studies were negative. Radiological studies such as CT scan and MRI were negative. In the setting of negative laboratory studies, imaging studies, and normal ENT and neurological evaluations, with symptoms unresponsive to conventional motion sickness therapy, a provisional diagnosis of MDS was made. The patient was started on low dose benzodiazepine (valium 2 mg at bedtime), which aided her nighttime symptoms. The patient received physical therapy for focused vestibular/balance rehabilitation, which offered partial relief. The patient joined the MDS online foundation support group, a forum to interact with other MDS patients and former patients who had recovered. She was carefully followed up in the outpatient until spontaneous resolution of symptoms after 8-9 months and she is completely back to baseline.

## 3. Discussion

MDS is a disorder of false perception of movement, its true incidence is unknown; its true etiology is also unknown [10]. Hypotheses such as release of information, which has been stored as vestibular information from the hippocampus and increased metabolism in the entorhinal cortex and amygdala, may be plausible [7, 11]. This may lead to overprocessing and storage of spatial information with less downregulation, poor recalibration, and readaptation [7, 10–12]. A study described a reduction of income in some study participants as being significant; the economic loss of MDS is also significant. Quality of life especially as related to concentration and financial resources are affected [8, 9, 13].

Females are more affected than males, especially women in their 4th decade of life [14]. Symptoms include rocking, bobbing, swaying, and floating. Patients feel unsteady and imbalanced; however they may return to baseline when they are back in motion or are exposed to their prior environment [1–20]. Patients typically do not complain of head trauma, and there is no family history of similar condition [20]. MDS is a diagnosis of exclusion [20]. Patients are previously healthy and have been exposed to long periods of travel in which passive motion is involved [18]. Many patients have been informed that they have anxiety disorders and depression based on their manifestations instead of being informed that their issues are the result of MDS [19, 20]. Benzodiazepines, amitriptyline, and physical therapy have proven beneficial [1–20].

A thorough history, physical examination, and diagnostic workup will be required to make a diagnosis; however nothing will be revealed after extensive workup [1]. If this syndrome is well comprehended, it may be more appropriately diagnosed [1].

Our patient presented with the classical symptoms and history of MDS and based on negative findings and the spontaneous resolution of her condition, MDS was the most plausible diagnosis. For patients who continue to demonstrate persistence of symptomatology, there is a tendency that resolution may be unlikely [14]. Depression and anxiety are not related to the condition even though some MDS patients may present with these conditions. They are a consequence of debilitation, our patient however did not present with these conditions [14]. This condition may or may not be associated with tiredness, headache, disorientation, anxiety, and depression [1]. Some sufferers of the condition complain about waves pushing them to one side [6]. Vestibuloocular reflex (VOR) is maladapted and symptoms may be reduced by readaptation of the VOR [2]. The cause of this condition is not well understood. So many theories exist in the literature about the etiology however; the condition may be a disorder of neuroplasticity especially because symptoms do not appear to respond to vestibular therapy [10]. Most patients would have their symptoms resolve spontaneously however; patients with this condition are managed with benzodiazepines and physical therapy as well as support groups [6, 20]. Follow-up with these patients as well as the involvement of the ENT physicians and neurologists may become necessary.

## 4. Conclusion

It is important to consider MDS in a patient with the following: a patient who has recently returned from a trip (land, air, or boat) with complaints of abnormal perception of motion who also has negative findings on clinical evaluation as well as negative laboratory findings and radiological tests. If the diagnosis is missed, morbidity could worsen and depression and anxiety may ensue. The key to successful management of patients with MDS is recognizing the condition with its classical presentation, reassurance, benzodiazepines, and support.
